# Using reverse genetics tool for study of Senecavirus A: pros and cons

**DOI:** 10.3389/fvets.2025.1546709

**Published:** 2025-04-02

**Authors:** Lijie Zhu, Zhijuan Li, Yan Li, Fuxiao Liu, Shengnan Yang

**Affiliations:** ^1^College of Veterinary Medicine, Qingdao Agricultural University, Qingdao, China; ^2^Qingdao Center for Animal Disease Control and Prevention, Qingdao, China

**Keywords:** *Senecavirus* A, reverse genetics, cDNA clone, rescue, fluorescent marker

## Introduction

*Senecavirus* A (SVA) is an emerging virus, typically inducing a vesicular disease in pigs. Clinical signs are characterized by lethargy and lameness, usually followed by the development of vesicles on the snout, dewclaw or (and) coronary band. The clinical signs are generally indistinguishable from those of other vesicular diseases in pigs (1). SVA-infected cases have been reported in at least 10 countries (2–11), therefore attracting a great deal of attention from the pig industry worldwide.

**Figure 1:**
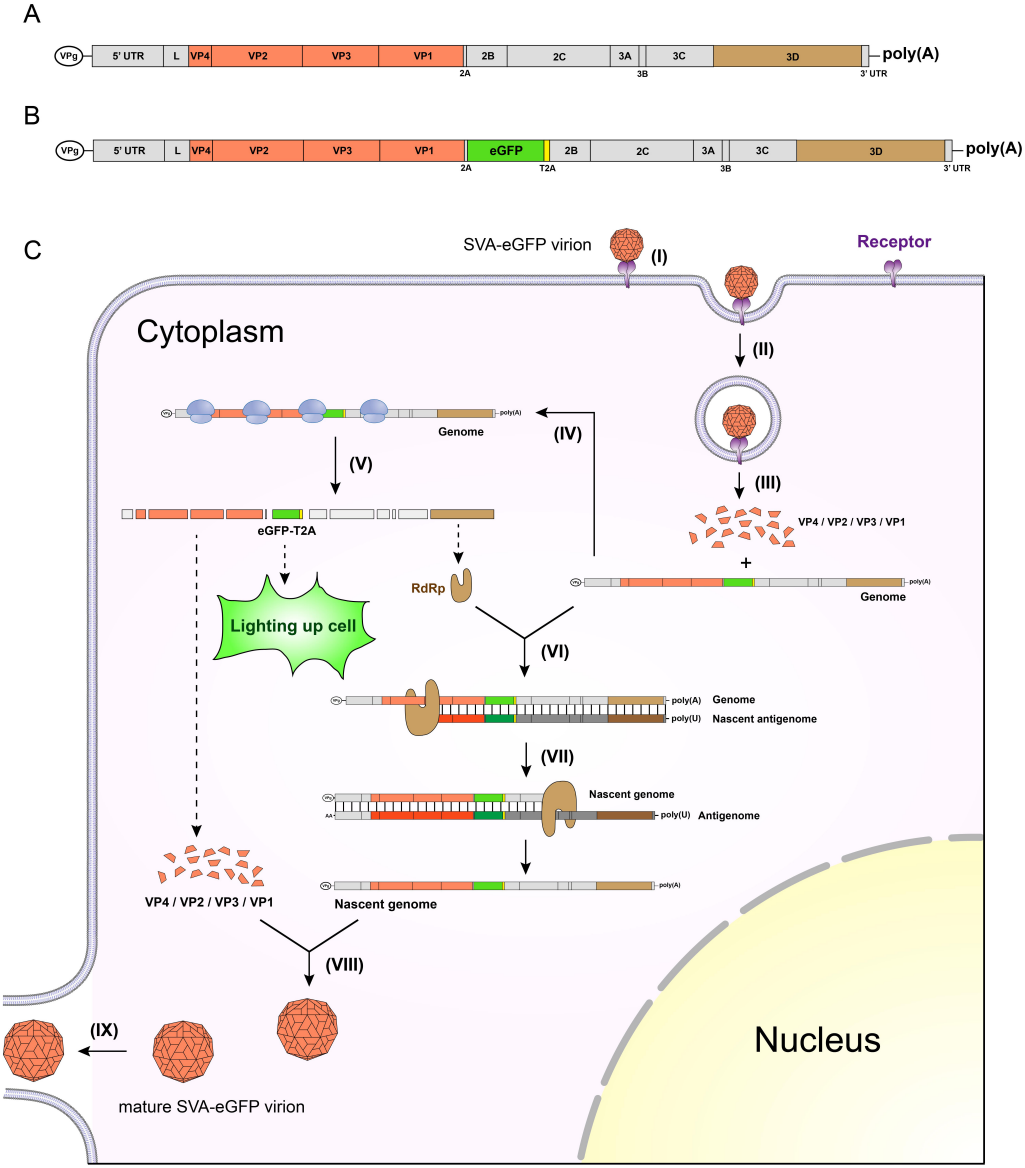
Schematic representations of wild-type genome, chimeric genome, and a single round of replication cycle after SVA-eGFP infection. Wild-type **(A)** and chimeric **(B)** SVA genomes. The latter contains an eGFP-T2A fusion fragment, located between the viral 2A and 2B sequences. Genetic elements match their actual lengths in proportion. A single round of viral replication cycle in a cell infected with SVA-eGFP **(C)**. The replication cycle mainly includes nine steps, namely, (I) virus attaching to its receptor, (II) endocytosis-mediated entrance of virion into cell, (III) uncoating of virion, (IV) ribosome-triggered translation of polyprotein, (V) further processing of polyprotein through lysis, (VI) RdRp-initiated replication of nascent antigenome, (VII) replication of nascent genome, (VIII) morphogenesis of virion, and (IX) release of virion.

SVA is the only member of the genus *Senecavirus*, in the family *Picornaviridae* (12). The virion is a typical icosahedral particle, encapsulating its own genome that is a positive-sense, single-stranded RNA genome (Figure 1A), approximately 7,300 nt in length, composed of 5' untranslated region (UTR), polyprotein open reading frame (ORF) and 3' UTR. SVA has a length-variable poly(A) tail at the genomic 3' terminus (13). At the 5' terminus, there is no cap structure. Alternatively, a short peptide, VPg, is covalently linked to the 5' terminus, and as the protein primer, plays a crucial role in the synthesis of viral genome.

In an SVA-infected cell, the viral polyprotein precursor is translated and progressively cleaved into 12 proteins: L, VP4, VP2, VP3, VP1, 2A, 2B, 2C, 3A, 3B, 3C and 3D (14). The VP1 to VP4 are structural proteins, which interact with one another, responsible for the viral morphogenesis. The others are non-structural proteins, required for viral replication (15–17). For example, the 2A oligopeptide confers a ribosome “skipping” effect, separating two proteins without needing a proteinase (18).

## Construction of SVA reverse genetics platform: general methodology

The reverse genetics (RG) technique is a potent tool, whereby a replication-competent RNA virus can be rescued (or recovered) from its genome cDNA clone, facilitating studies on molecular virology, virus-vectored vaccinology, marker-tagged diagnostics and so forth. To data, several groups have independently constructed the RG platform of SVA, using their individual methodologies (19–25), slightly different from one another. We have established an SVA RG platform, demonstrated to be highly efficient in the recovery of viable SVA (26).

The first step, taking our method to develop the SVA RG system as an example, was to construct a cDNA clone of full-length genome, through either chemical synthesis or overlap extension PCR. The cDNA clone was flanked by a T7 promoter and a poly(A) tail at its 5' and 3' ends, respectively. The fusion sequence, T7 promoter-cDNA clone-poly(A) tail, was subcloned into a plasmid for constructing a recombinant one. For virus rescue, BSR-T7/5 cells, which could constitutively express the T7 RNA polymerase, were seeded onto a 6-well plate for incubation overnight at 37°C, followed by transfection with the cDNA clone-containing plasmid. The plasmid-transfected cell monolayer was subsequently cultured at 37°C for 3 d, and then subjected to two freeze-and-thaw cycles to harvest the supernatant for serial blind passages. The passage-5 progeny was analyzed by RT-PCR for detecting whether the virus of interest was successfully rescued.

## RG-generated wild-type SVA for omics studies

SVA can be clinically isolated from SVA-infected pigs. An SVA isolate, if not subjected to several rounds of plaque purification, may not be a single SVA clone, even containing other pathogens in the viral stock. If so, an SVA-based omics study would be invaluable. In contrast, RG-generated SVA is a single strain without contamination by other pathogens, required for a comparative study of omics after SVA infection. In our previous study, a wild-type SVA was successfully rescued from its cDNA clone (26). The passage-5 progeny was inoculated into cell monolayers, followed by a series of comparative analyses based on multi-omics, including transcriptomics (27), proteomics (28) and metabolomics (29).

## RG-mediated point mutation for unveiling SVA characteristics

It has been widely reported that one single point mutation can even extensively change the picornaviral characteristics (30–33). Site-directed mutagenesis (SDM) can be used for modifying a wild-type SVA cDNA clone to reconstruct a mutant. If able to be rescued from the mutant, the SDM-modified SVA can be compared with its wild-type counterpart concerning some viral features. For example, two SVA variants were constructed *via* SDM to introduce different mutated sites in their RdRps (S460L alone and I212V-S460L in combination), consequently suggesting both variants that had lower recombination capacity than the wild-type strain (34). More recently, Zhao et al. (13) used the RG tool with method of structural biology to optimize novel oncolytic SVA mutants: viral receptor-associated SVA-S177A, and viral antigenic peptide-related SVA-S177A/P60S, both of which showed not only higher infectivity but also lower immunogenicity than the wild-type strain did (35).

## Fluorescent marker-tagged SVA facilitating viral studies

Picornavirus is capable to accommodate a foreign sequence in its genome. The shorter the foreign sequence is, the stronger the accommodation capability is. Fluorescent protein and luciferase sequences are widely used as tracking markers inserted into wild-type SVA cDNA clones to rescue marker-tagged viruses for antiviral screening, virus neutralization test, oncolytic analysis, *etc* (19, 25, 36, 37). These fluorescent markers include enhanced green fluorescent protein (eGFP), red fluorescent protein, NanoLuc^®^ luciferase (NLuc), *Gaussia* luciferase, and so on.

### eGFP-tagged SVA

The eGFP is most broadly used for constructing fluorescent marker-tagged picornaviruses. An eGFP-tagged SVA (SVA-eGFP) has a chimeric genome that generally contains an eGFP ORF fused with a *Thosea asigna* virus 2A (T2A) sequence (Figure 1B). The SVA-eGFP virion can enter a susceptible cell *via* an endocytic pathway, followed by a series of processes to encode the eGFP and finally to generate mature virions in the cell, as schematically shown in Figure 1C. Notably, the eGFP is expressed as the eGFP-T2A fusion pattern, which can “light up” the cell through observation using a fluorescence microscope, indicating that the cell has been infected with SVA-eGFP.

Viral titration based on TCID_50_ assay is involved in observing cytopathic effect (CPE) for calculating a titer value. However, a few reports have shown that some SVA strains hardly induce the typical CPE on a cell monolayer (7, 38, 39), therefore inconducive to the viral titration. One of the advantages in use of SVA-eGFP is facilitating the viral titration, because fluorescence-emitted wells in the TCID_50_ assay indicate that their corresponding cell monolayers have been infected with SVA-eGFP. The accuracy of titer measurement through identifying fluorescent wells is much higher than that through observing the CPE formation.

The conventional virus neutralization test (VNT) is generally considered as a gold standard for recognizing virus-specific antibodies, but unfortunately is a labor- and time-consuming method. If a wild-type SVA is used for VNT, the prerequisite is that the wild-type SVA is able to induce obvious CPE on a 96-well plate. In contrast, if SVA-eGFP is used, CPE would be unnecessary for VNT readings, because the SVA-eGFP can function as a virus-infected indicator to imply which wells are infected or not. Fluorescence-free wells suggest that neutralizing antibodies have completely neutralized SVA-eGFP at a certain dilution of viral suspension. More importantly, 2 days are enough for the incubation of 96-well plate to recognize a fluorescent phenotype after SVA infection, whereas the wild-type SVA-based VNT need at least 4 days. Therefore, use of SVA-eGFP contributes to VNT for clinical sera (19, 25).

Another SVA-eGFP-related advantage is favorable to determine whether a replication-competent virus is successfully rescued from its cDNA clone. If there is no green fluorescence with serial blind passages, commonly three passages after plasmid transfection, it can be concluded that the viable SVA-eGFP fails to be rescued. This is very important especially for a large number of genetically modified SVA-eGFP cDNA clones that need to be analyzed for uncovering their own recovery abilities. For example, Meng et al. (40) constructed a series of 36 different cDNA clones, independently transfected into cell monolayers in an attempt to rescue genetically modified SVA-eGFPs. The green fluorescence was demonstrated to facilitate the fast screening for some SVA-eGFP cDNA clones without ability of virus recovery (40).

### Luciferase-tagged SVA

Luciferases are proteins with enzymatic activity, which can catalyze the oxidation of substrate (commonly luciferin), causing the emission of photons. Luciferase is another fluorescent marker, widely used for the construction of recombinant viruses using RG technique. Different from fluorescent proteins, on the one hand, luciferases do not need the excitation light for lighting up cells but require appropriate luciferins; on the other hand, luciferase-induced fluorescence can be quantified using a microplate reader, whereas fluorescent protein-induced fluorescence cannot be done.

There are various types of luciferases with their own characteristics. NLuc is a novel luciferase, shorter, smaller and “brighter” than the other two common ones, firefly and *Renilla reniformis* luciferases. NLuc is a promising fluorescent marker to construct chimeric viruses for imaging assay *in vitro* or *in vivo* (41–44). The NLuc-tagged SVA (SVA-NLuc) is able of efficiently expressing NLuc, which as a bright indicator has been demonstrated to facilitate greatly the SVA-mediated oncolytic analysis *in vitro* (37), whereas there has unfortunately been no further study on an *in vivo* test.

The luminescence intensity is roughly proportional to the titer of luciferase-tagged SVA in cells. Such a quantifiable feature contributes to the high-throughput screening of anti-SVA drugs (36, 45, 46). For example, Wang et al. (23) used the SVA-NLuc to analyze a drug library composed of 136 natural products, and rapidly screened out five efficient anti-SVA products, namely, monensin sodium salt, progesterone, hypophyllanthin, 4-hydroxyderricin, and 2-methoxyestrone (45). In addition, its quantitative trait significantly shortens the period of conventional method for determining an SVA growth curve (37).

## Drawback of marker-tagged SVAs: genetic instability with passaging

As mentioned above, marker-tagged SVAs greatly facilitate studies of SVA, whereas unfortunately, foreign sequences have been demonstrated to be genetically unstable in recombinant SVAs. We found that SVA-eGFP-induced fluorescence was gradually weakening in intensity with viral passaging, and RT-PCR demonstrated that the eGFP sequence was consecutively deleted from the chimeric SVA genome (19). The eGFP ORF is 720 bp in length, longer than the NLuc ORF (516 bp). In theory, the longer a marker is, the more unstable its sequence is. Indeed, we found that NLuc was genetically more stable than eGFP with passaging (37), whereas, as a foreign fragment, the NLuc sequence was still deleted gradually from the SVA-NLuc genome with passaging. Such an unstable feature limits the application of marker-tagged SVAs, resulting in only low-passage progenies that can be used.

## Development of novel SVA vaccine using RG technique

Because a foreign sequence is unstable in a chimeric SVA genome, SVA has no potential in developing a virus-vectored vaccine. In addition, different SVA strains are prone to genetic recombination with each other (47). Therefore, live vaccines cannot be used in preventing SVA infection. Conventional inactivated vaccines have been widely reported to be safe and efficient against SVA infection (39, 48–52). Interestingly, Fan et al. (53) used the RG tool to construct an engineering His-tagged SVA, further processed into a novel inactivated vaccine. In this study, one 6 × His tag was fused to the C-terminal of VP1, and could be displayed on the surface of SVA virion. The 6 × His tag was so short that it was relatively stable in the chimeric SVA with passaging, and more importantly, allowed one-step purification of SVA antigens through Ni^2+^ affinity columns (53). Such an engineering strain provided a novel method of concentrating SVA antigens for the production of inactivated vaccines.

## Future challenges for developing RG system of SVA

Numerous groups have established their own RG systems for SVA recovery (19–25), opening up new avenues for in-depth dissection of SVA molecular mechanisms, whereas some technique challenges still remain to be addressed. The first one is how a full-length cDNA clone is rapidly, efficiently constructed. We found that many genetically unstable fragments were possibly prone to being deleted from a longer sequence *via* the conventional method, restriction enzyme-based cloning, for constructing an SVA cDNA clone. Chemical synthesis, albeit costly, is more efficient than the conventional method in constructing a full-length cDNA clone. The second challenge is rescue of a strain that can induce obvious CPEs on a cell monolayer. We previously rescued different SVA strains from their own cDNA clones. Unfortunately, SVA-induced CPE was almost invisible during initial passaging *in vitro* (26), therefore limiting the application of SVA as a model virus. The third challenge is how a risk-free SVA strain is rescued as a model virus for use *in vivo*. In other words, we should use the RG technique to facilitate our studies on SVA, rather than to create some putative risks in bio-safety.
